# *NLRC4* gene silencing-dependent blockade of NOD-like receptor pathway inhibits inflammation, reduces proliferation and increases apoptosis of dendritic cells in mice with septic shock

**DOI:** 10.18632/aging.202379

**Published:** 2021-01-06

**Authors:** Shi-Sheng Wang, Chun-Song Yan, Jun-Ming Luo

**Affiliations:** 1 Department of Respiratory Medicine, The Second Affiliated Hospital of Nanchang University, Nanchang 330006, P.R. China

**Keywords:** *NLRC4*, inflammatory reaction, immune response, NOD-like receptor pathway, septic shock

## Abstract

Septic shock is one of the most significant health concerns across the world, involving hypo-perfusion and defects in tissue energy. The current study investigates the role of NLR family CARD domain containing protein 4 (*NLRC4)* in septic shock-induced inflammatory reactions, lung tissue injuries, and dendritic cell (DC) apoptosis. Septic shock mice models were established by modified cecal ligation and puncture and injected with retroviral vector expressing siRNA-*NLRC4*. DCs were then isolated and transfected with siRNA-*NLRC4*. The degree of lung tissue injury, cell cycle distribution, cell apoptosis and cell viability of DCs were assessed. *NLRC4* was found to be expressed at high levels in mice with septic shock. *NLRC4* silencing inhibited the activation of the NOD-like receptor (NLR) pathway as evidenced by the decreased levels of *NOD1*, *NOD2*, *RIP2*, and *NF-κB*. In addition, *NLRC4* silencing reduced the inflammatory reaction as attributed by reduced levels of IL-1β, TNF-α and IL-6. Suppressed *NLRC4* levels inhibited cell viability and promoted cell apoptosis evidenced by inhibited induction of DC surface markers (CD80, CD86, and MHC II), along with alleviated lung tissue injury. In conclusion, *NLRC4* silencing ameliorates lung injury and inflammation induced by septic shock by negatively regulating the NLR pathway.

## INTRODUCTION

According to the International Guidelines of the Surviving Sepsis Campaign, septic shock is characterized by sepsis-induced persistent hypotension, in spite of sufficient fluid resuscitation [1]. As a chief source of infection, severe sepsis and septic shock with sepsis-related multiple organ failure account for a significant proportion of mortality in intensive care units (ICUs) even in developed countries [2], with the published mortality rate among hospitals ranging between 25% and 70% on a global scale. Based on the widely-accepted concepts involving sepsis and septic shock, its clinical manifestations occur as a result of interactions between inflammatory, coagulation pathways and infectious pathogens [3]. Although the rates of septic shock-related mortality have exhibited a gradual downtrend largely owing to increased awareness, careful attention, improved early antibiotic treatment and the discovery of hemodynamic indicators, there are still no specific drug treatment regimens for patients who develop the condition [4]. Genetic epidemiology suggests that there is an element of susceptibility in relation to an individual’s genetic make-up, resulting in significant differences in responses to infection, thus posing an added degree of complexity for clinicians [5]. Therefore, the aim of this study was to explore the genetic influence of nucleotide oligomerization domain (NOD)-like receptor (NLR) family CARD domain containing protein 4 (*NLRC4*) on the treatment of septic shock by mediating the NLR pathway.

Genetic factors play a major role in the mortality of patients with septic shock caused by infections [2]. Reports have documented multiple over-expressed genes in innate immunity, including chemokine receptor, cytokine, and toll-receptor pathways [6]. The gene silencing paradigm is embedded in a larger framework of genetic recombination, and is implicated in the generation of functional and clinical phenotypes in certain inflammatory diseases [7]. Over a decade ago, *NLRC4* was identified and regarded as a member of the NLR family of intracellular sensors [8]. Furthermore, another member of the NLR family, the *NLRP3* inflammasome, has been regarded as a chief component of the innate immune system in the identification of viral infections [9]. The dysregulation of the innate immune system is well-known to serve as a crucial factor in the onset of sepsis, combined with genetic variability as a potential targeted therapy for sepsis and septic shock [5]. The innate immune system is also characterized by dendritic cell (DC) loss and contributes to dismal outcomes or nosocomial infections [10]. Therefore, this study aimed at investigating the effects of *NLRC4* gene silencing on inflammatory reactions, shock-induced lung tissue injuries, and DC-mediated immune responses in septic shock mice models by mediating the NLR pathway.

## RESULTS

### *In silico* analysis of NLRC4 in septic shock

**Figure 1:**
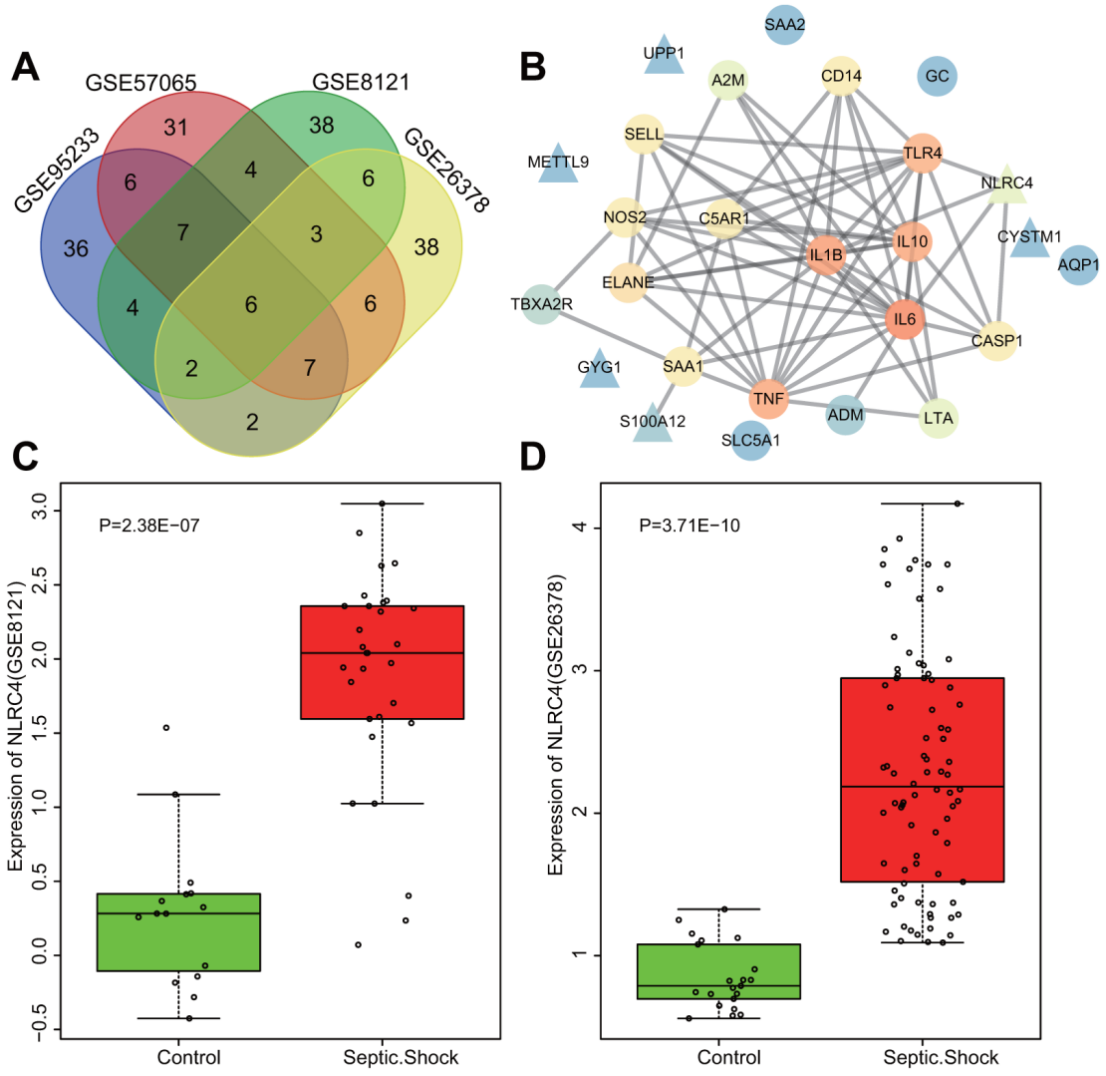
**Highly expressed *NLRC4* was found in septic shock.** (**A**) 6 intersected genes detected among the top 70 DEGs from the microarray expression profiles of GSE95233, GSE57065, GSE8121 and GSE26378. (**B**) PPI network of the DEGs and septic shock-related genes (the triangle signifies the DEGs, the circle indicates the septic shock-related genes; the color of the genes shows the correlation degree with other genes, with orange coloration indicative of a high correlation degree and a blue coloration signifying a low correlation degree). (**C**, **D**) The expression of *NLRC4* in septic microarray expression profiles GSE8121 and GSE26378.

Datasets regarding septic shock obtained from the gene expression omnibus (GEO) database (https://www.ncbi.nlm.nih.gov/geo/) were differentially analyzed, with adj.P.Val < 0.05 and |LogFoldChange| > 2 as the screening threshold. A total of 1123, 864, 416 and 585 differentially expressed genes (DEGs) were retrieved from the GSE95233, GSE57065, GSE8121 and GSE26378 datasets (Table 1) respectively, among which the top 70 DEGs with the largest fold change were selected from each microarray for intersectional analysis with a Venn diagram (http://bioinformatics.psb.ugent.be/webtools/Venn/) (Figure 1A). The diagram highlighted 6 intersected genes (*S100A12*, *UPP1*, *GYG1*, *NLRC4*, *METTL9*, and *CYSTM1*) as candidates for further investigation. Additionally, disease genes associated with septic shock were retrieved from the DisGeNET database (http://www.disgenet.org/web/DisGeNET/menu/search?4), with the top 20 genes (*TNF*, *C5AR1*, *GC*, *AQP1*, *NOS2*, *SELL*, *TBXA2R*, *A2M*, *ELANE*, *IL6*, *IL10*, *TLR4*, *CASP1*, *ADM*, *IL1B*, *LTA*, *CD14*, *SAA1*, *SAA2* and *SLC5A1*) considered to be septic shock-related genes. The interaction between septic shock-related genes and DEGs was analyzed using the String database (https://string-db.org/). The PPI net was produced via Cytoscape 3.6.0 software [11] (Figure 1B), wherein *NLRC4* was discovered to be closely associated with several septic shock-related genes, signifying association with the occurrence of septic shock. Supplementary Figure 1A, 1B illustrate the heat maps of the top 70 DEGs in the microarrays of GSE95233 (Figure 1C) and GSE57065 (Figure 1D). Moreover, higher expression of *NLRC4* was observed among patients with septic shock compared to healthy individuals. *NLRC4* is a member of the NLR family that is involved in the activation of the NLR pathway associated with inflammatory reactions [12, 13]. Previous reports have demonstrated that *NOD1* and *NOD2* exert significant roles in septic shock [14, 15], yet the exact extent of the influence and mechanism of *NLRC4* in septic shock still remains unclear. Given the above bioinformatics analysis and preliminary findings, we suspected that abnormal expressions of *NLRC4* might influence the NLR pathway in septic shock.

**Table 1:** Baseline information of septic shock expression profiles GSE95233, GSE57065, GSE8121 and GSE26378.

**Accession**	**Platform**	**Organism**	**Tissue**	**Sample**
GSE95233	GPL570	Homo sapiens	Blood	51 septic shock patients and 22 healthy volunteers
GSE57065	GPL570	Homo sapiens	Whole blood	28 septic shock patients and 25 healthy volunteers
GSE8121	GPL570	Homo sapiens	Whole blood	15 normal children and 30 children with septic shock
GSE26378	GPL570	Homo sapiens	Whole blood	82 children with septic shock and 21 normal controls

### NLRC4 silencing alleviates lung tissue injury induced by septic shock

**Figure 2:**
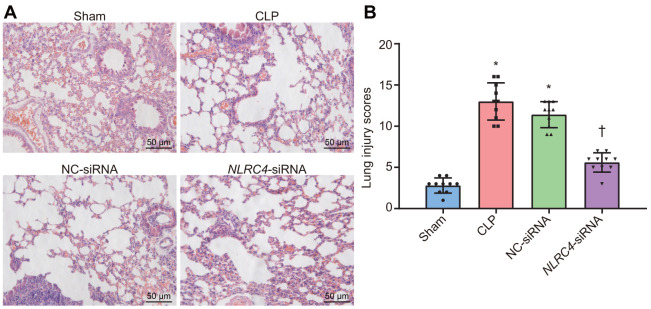
**HE staining results illustrate the amelioration of lung tissue injury induced by septic shock in mice after DCs transfected with *NLRC4*-siRNA.** (**A**) The lung tissue injury detected by HE staining (scale bar = 50 μm). (**B**) The lung tissue injury scores. n = 10. * *p* < 0.05 vs. the sham group. † *p* < 0.05 vs. the CLP and NC-siRNA groups. Data comparison among multiple groups was analyzed by one-way analysis of variance (ANOVA), followed by Tukey’s post hoc test. The experiment was repeated 3 times independently.

Lung tissue injury induced by septic shock was assessed after DCs were transfected with *NLRC4*-siRNA. Hematoxylin and eosin (HE) staining results (Figure 2A) revealed that in the sham group, the lobule-alveolar structure in the mouse lung tissue was intact with mild widened-gaps without fiber tissue hyperplasia or inflammatory cell exudation in the alveolar spaces. The mice in the cecal ligation and puncture (CLP) and negative control (NC)-siRNA groups presented with symptoms and signs of alveolar congestion, hemorrhage, alveolar wall thickening, fracture and collapse in most of the mouse lung tissue. Large amounts of inflammatory cells were also detected in the alveoli lumen and interstitial tissues of lungs. In the *NLRC4*-siRNA group, mice displayed intact alveolar structures in the lungs, with the presence of mild widened-gaps but without hemorrhage, well-preserved fibrous structure, or inflammatory exudate in the alveolar spaces. Lung tissue injury scoring revealed that the CLP and NC-siRNA groups received significantly higher lung tissue injury scores compared to that of the sham group. In comparison, significantly lower lung tissue injury scores were observed in the *NLRC4*-siRNA group relative to that of the CLP and NC-siRNA groups (Figure 2B). Based on the aforementioned observations, we concluded that *NLRC4* gene silencing could alleviate lung tissue injury induced by septic shock.

### Silencing of NLRC4 inhibits the NLR pathway by down-regulating the expressions of NOD1, NOD2, RIP2 and NF-κB in mice lung tissues

After HE staining, the mRNA and protein expression of NLR pathway-related genes in mice’s lung tissues were quantified. Reverse transcription quantitative polymerase chain reaction (RT-qPCR) results revealed that the CLP and NC-siRNA groups presented with significantly elevated mRNA expressions of *NLRC4, NOD1, NOD2, RIP2*, and *NF-κB* when compared to the sham group (all *p*_sham vs. CLP_ < 0.001; *p*_sham vs. NC-siRNA_ < 0.001), with no notable changes detected regarding expressions of *NLRC4* (*p*_sham vs. *NLRC4*-siRNA_ = 0.334), *NOD1* (*p*_sham vs. *NLRC4*-siRNA_ = 0.425), *NOD2* (*p*_sham vs. *NLRC4*-siRNA_ = 0.964), *RIP2* (*p*_sham vs.*NLRC4*-siRNA_ = 0.367) or *NF-κB* (*p*_sham vs. *NLRC4*-siRNA_ = 0.414) in the *NLRC4*-siRNA group. In comparison to the CLP and NC-siRNA groups, the *NLRC4*-siRNA group displayed markedly diminished mRNA expressions of *NLRC4, NOD1, NOD2, RIP2*, and *NF-κB* (all *p*_CLP vs. *NLRC4*-siRNA_ < 0.001; *p*_NC-siRNA vs. *NLRC4*-siRNA_ < 0.001). These results indicated that *NLRC4* gene silencing functioned to block the NLR pathway.

**Figure 3:**
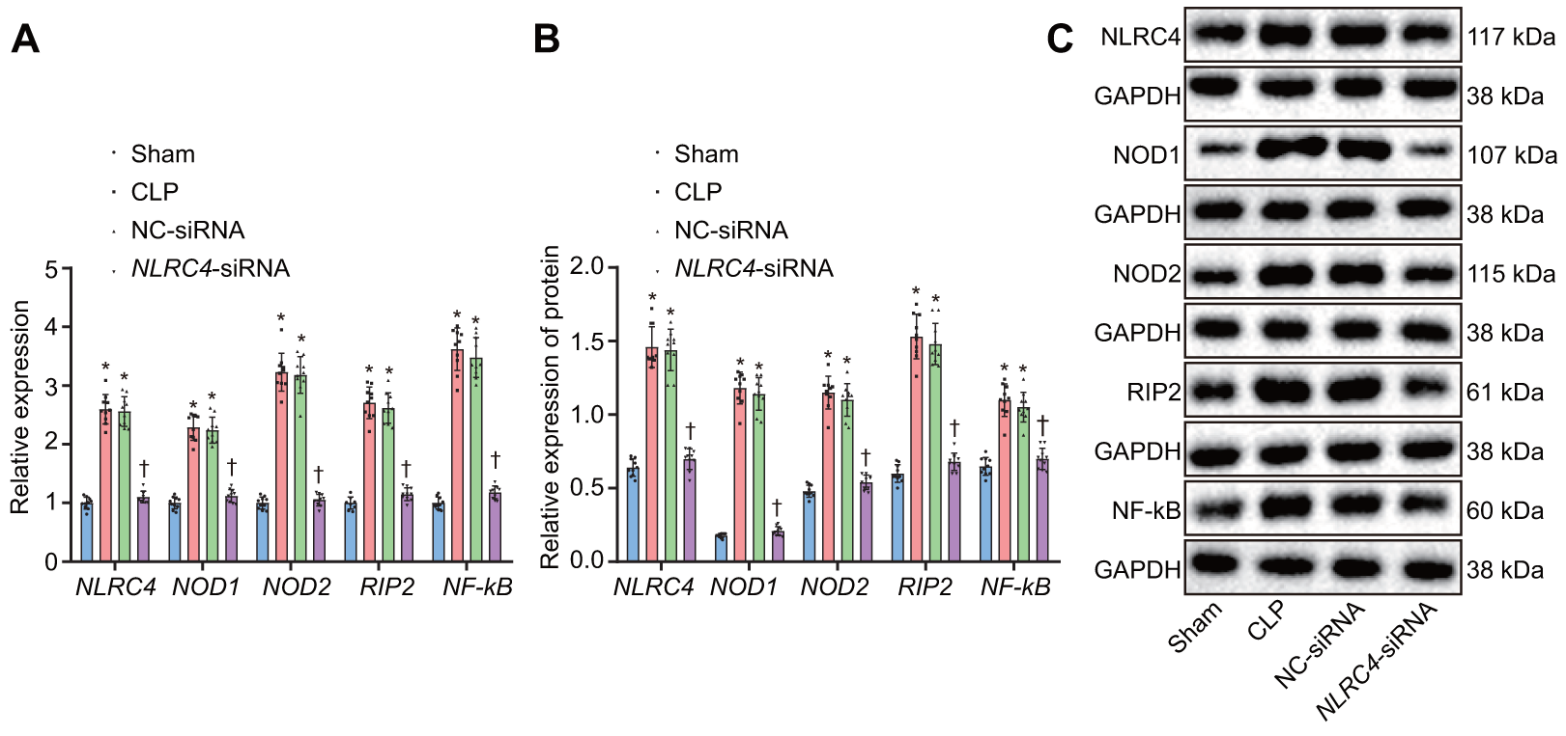
**Silencing of *NLRC4* inactivates the NLR pathway in mouse lung tissues.** (**A**) The mRNA expression of *NLRC4*, *NOD1*, *NOD2*, *RIP2*, and *NF-κB* in DCs determined by RT-qPCR. (**B**) The protein expression of NLRC4, NOD1, NOD2, RIP2, and NF-κB normalized to GAPDH in DCs measured by Western blot analysis. (**C**) Western blot bands of NLRC4, NOD1, NOD2, RIP2, and NF-κB in different transfection groups. N = 10. * *p* < 0.05 vs. the sham group. † *p* < 0.05 vs. the CLP and NC-siRNA groups. Data comparison among multiple groups was analyzed by one-way ANOVA, followed by Tukey’s post hoc test. The experiment was repeated 3 times independently.

Western blot analysis results elaborated that the CLP and NC-siRNA groups exhibited significantly increased protein expressions of NLRC4, NOD1, NOD2, RIP2, and NF-κB compared to that of the sham group (all *p*_sham vs. CLP_ < 0.001; *p*_sham vs. NC-siRNA_ < 0.001), while no changes were detected regarding protein expressions of NLRC4 (*p*_sham vs. *NLRC4*-siRNA_ = 0.636), NOD1 (*p*_sham vs. *NLRC4*-siRNA_ = 0.124), NOD2 (*p*_sham vs. *NLRC4*-siRNA_ = 0.398), RIP2 (*p*_sham vs. *NLRC4*-siRNA_ = 0.389), or NF-κB (*p*_sham vs. *NLRC4*-siRNA_ = 0.574)in the *NLRC4*-siRNA group. In contrast with the CLP and NC-siRNA groups, remarkably declined protein levels of NLRC4, NOD1, NOD2, RIP2, and NF-κB were noted in the *NLRC4*-siRNA group (*p*_CLP vs. *NLRC4*-siRNA_ < 0.001; *p*_NC-siRNA vs. *NLRC4*-siRNA_ < 0.001). These results further verified that *NLRC4* gene silencing blocked the NLR pathway (Figure 3A–3C).

### NLRC4 gene silencing alleviates inflammatory reaction and reduces inflammatory cell infiltration

**Figure 4:**
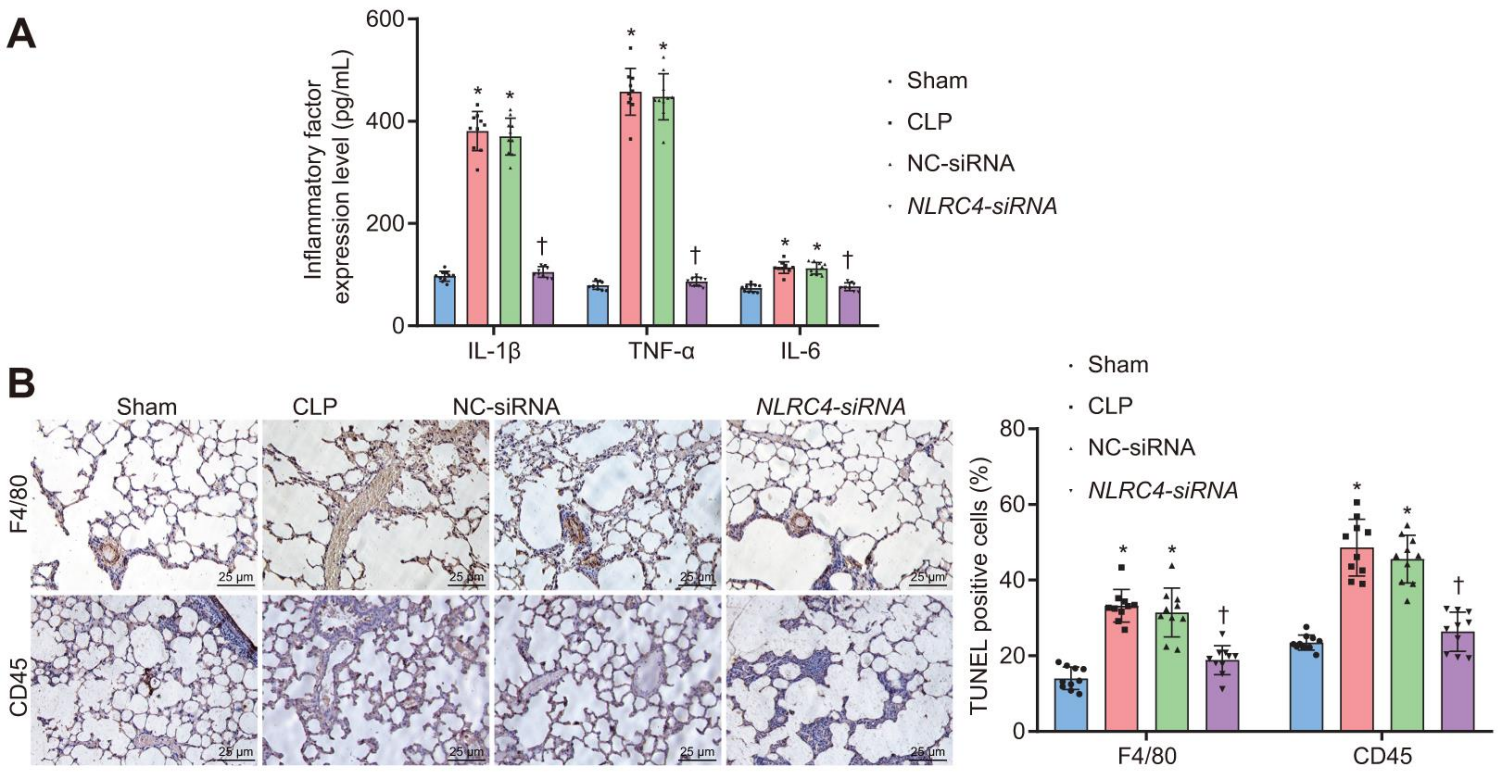
***NLRC4* downregulation alleviates inflammatory reaction and reduces inflammatory cell infiltration.** (**A**) the levels of IL-1β, TNF-α and IL-6 in lung tissues of mice with septic shock detected by ELISA. (**B**) inflammatory cell infiltration evidenced by F4/80 and CD45 positive levels using TUNEL staining (scale bar = 25 μm). n = 10. * *p* < 0.05 vs. the sham group. † *p* < 0.05 vs. the CLP and NC-siRNA groups. Data comparison among multiple groups was analyzed by one-way ANOVA, followed by Tukey’s post hoc test. The experiment was repeated 3 times independently.

Enzyme-linked immunosorbent assay (ELISA) was performed on septic mice after their DCs were transduced with different vectors to evaluate the effects of *NLRC4* gene silencing on the inflammatory reaction of septic shock. Results (Figure 4A) demonstrated that the CLP and NC-siRNA groups exhibited markedly elevated levels of Interleukin (IL)-1β, tumor necrosis factor α (TNF-α) and IL-6 compared to that of the sham group (all *p*_sham vs. CLP_ < 0.001; *p*_sham vs. NC-siRNA_ < 0.001), while no remarkable differences were observed in the expression levels of IL-1β (*p*_sham vs. *NLRC4*-siRNA_ = 0.085), TNF-α (*p*_sham vs. *NLRC4*-siRNA_ = 0.382) or IL-6 (*p*_sham vs. *NLRC4*-siRNA_ = 0.889) in the *NLRC4*-siRNA group. Reduced levels of IL-1β, TNF-α and IL-6 were detected in the *NLRC4*-siRNA group, compared with the sham and NC-siRNA groups (all *p*_CLP vs. *NLRC4*-siRNA_ < 0.001; *p*_NC-siRNA vs. *NLRC4*-siRNA_ < 0.001), which suggested that *NLRC4* gene silencing alleviated the inflammatory reaction induced by septic shock. As reflected by Figure 4B, immunohistochemistry results revealed that the CLP and NC-siRNA groups exhibited markedly elevated levels of F4/80 (*p*_sham vs. CLP_ < 0.001; *p*_sham vs. NC-siRNA_ < 0.001) and CD45 (*p*_sham vs. CLP_ < 0.001; *p*_sham vs. NC-siRNA_ < 0.001) compared to the sham group (all *p*_sham vs. CLP_ < 0.001; *p*_sham vs. NC-siRNA_ < 0.001), whereas no marked changes were seen on the levels of F4/80 (*p*_sham vs.NLRC4-siRNA_ = 0.602) and CD45 (*p*_sham vs. NLRC4-siRNA_ = 0.619) in the *NLRC4*-siRNA group. The levels of F4/80 (*p*_CLP vs. NLRC4-siRNA_ < 0.001; *p*_NC-siRNA vs. NLRC4-siRNA_ < 0.001) and CD45 (*p*_CLP vs. NLRC4-siRNA_ < 0.001; *p*_NC-siRNA vs. NLRC4-siRNA_ < 0.001) in the *NLRC4*-siRNA group were markedly reduced in comparison to that of the CLP and NC-siRNA groups. Thus, these results indicated that *NLRC4* gene silencing reduced infiltration of inflammatory cells.

### DCs exhibit positive morphological changes after being cultured and NLRC4 gene silencing inhibits DC maturation and proliferation

**Figure 5:**
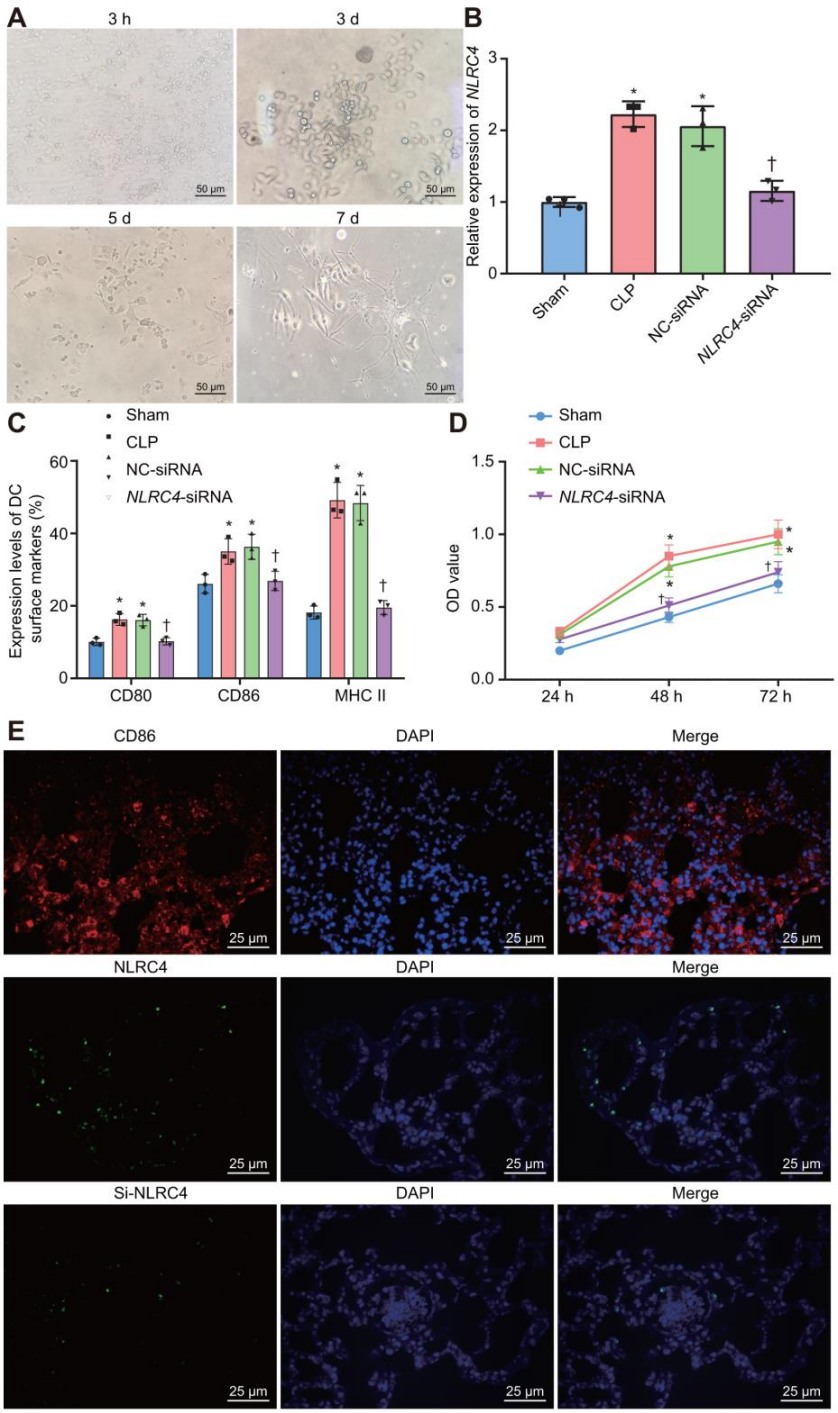
**Improved morphological changes in DCs after being cultured for 3 h, 3 d, 5 d, and 7 d and suppressed *NLRC4* reduces DC proliferation.** (**A**) Morphological changes of DCs observed under an inverted microscope (scale bar = 50 μm). (**B**) The expression of *NLRC4* in DCs determined by RT-qPCR. (**C**) The induction of DC surface markers, CD80, CD86, and MHC II, detected by flow cytometry. (**D**) The DC apoptosis as indicative of OD values detected by flow cytometry. (**E**) The immunofluorescence labeling of NLRC4 and DC surface marker CD86 (scale bar = 25 μm). The bone marrow-derived DCs from the same group of mice were adopted in the *in vivo* experiments. * *p* < 0.05 vs. the sham group. † *p* < 0.05 vs. the CLP and NC-siRNA groups. Data comparison among multiple groups was analyzed by one-way ANOVA, followed by Tukey’s post-hoc test. Data at different time points were compared by repeated measures ANOVA, followed by Bonferroni test. The experiment was repeated 3 times independently.

Morphological changes in DCs were observed under an inverted microscope. After 3 h of adherence, small round cells and a portion of the closely aggregated cells became apparent. On the 3^rd^ day after culture, a pattern of small densely arranged round cells were observed with a trivial amount of bud-shaped protuberances. On the 5^th^ day, adherent cells in dense arrangements were observed, with an increase in the bud-shaped protuberances. On the 7^th^ day, DCs appeared to be spindle shaped, closely-arranged with a large number of distinct protuberances (Figure 5A). RT-qPCR was employed to detect the *NLRC4* expression in DCs in each group (Figure 5B). The results demonstrated that *NLRC4* expressions were elevated in the CLP and NC-siRNA groups in contrast to that of the sham group (*p*_sham vs. CLP_ < 0.001; *p*_sham vs. NC-siRNA_ < 0.001), whereas the expressions of NLRC4 did not differ significantly in the *NLRC4*-siRNA group (*p*_sham vs. *NLRC4*-siRNA_ = 0.719). Significantly diminished *NLRC4* expressions were also detected in the *NLRC4*-siRNA group compared to that of the CLP and NC-siRNA groups (*p*_CLP vs. *NLRC4*-siRNA_ < 0.001; *p*_NC-siRNA vs. *NLRC4*-siRNA_ = 0.002). The findings illustrated that morphological changes in DCs induced by septic shock were improved by the silencing of *NLRC4*.

Furthermore, flow cytometry was utilized to investigate the influence of *NLRC4* gene silencing on the growth and maturation of DCs. The induction of DC surface markers [histocompatibility complex II (MHC II), CD80, and CD86] was detected. The results (Figure 5C) revealed that compared with the sham group, the induction of MHC II (*p*_sham vs. CLP_ < 0.001; *p*_sham vs. NC-siRNA_ < 0.001), CD80 (*p*_sham vs. CLP_ = 0.002; *p*_sham vs. NC-siRNA_ = 0.027) and CD86 (*p*_sham vs. CLP_ = 0.030; *p*_sham vs. NC-siRNA_ = 0.015) were all noticeably elevated in the CLP and NC-siRNA groups. However, no significant differences were detected regarding the induction of MHC II (*p*_sham vs. *NLRC4*-siRNA_ = 0.968), CD80 (*p*_sham vs. *NLRC4*-siRNA_ = 0.999) or CD86 (*p*_sham vs. *NLRC4*-siRNA_ = 0.988) in the *NLRC4*-siRNA group. In comparison with the CLP and NC-siRNA groups, reduced induction of MHC II (*p*_CLP vs. *NLRC4*-siRNA_ < 0.001; *p*_NC-siRNA vs. *NLRC4*-siRNA_ < 0.001), CD80 (*p*_CLP vs. *NLRC4*-siRNA_ = 0.025; *p*_NC-siRNA vs. *NLRC4*-siRNA_ = 0.031) and CD86 (*p*_CLP vs. *NLRC4*-siRNA_ = 0.047; *p*_NC-siRNA vs. *NLRC4*-siRNA_ = 0.023) were observed in the *NLRC4*-siRNA group. These results indicated that *NLRC4* gene silencing suppressed the maturation of DCs.

Lastly, 3-(4,5-Dimethyl-2-Thiazyl)-2,5-Diphenyl-2H-Tetrazolium Bromide (MTT) assay was performed to detect the effects of *NLRC4* gene silencing on DC proliferation. The results (Figure 5D) revealed that the cell proliferation ability was enhanced in the CLP and NC-siRNA groups compared to that of the sham group (48 h: *p*_sham vs. CLP_ < 0.001; *p*_sham vs. NC-siRNA_ < 0.001; 72 h: *p*_sham vs. CLP_ < 0.001; *p*_sham vs. NC-siRNA_ < 0.001), while no notable discrepancies in proliferation ability were detected in the *NLRC4*-siRNA group (48 h: *p*_sham vs. *NLRC4*-siRNA_ = 0.394; 72 h: *p*_sham vs. *NLRC4*-siRNA_ = 0.394). When compared with the CLP and NC-siRNA groups, significantly decreased cell proliferation ability was noted in the *NLRC4*-siRNA group (48 h: *p*_CLP vs. *NLRC4*-siRNA_ < 0.001; *p*_NC-siRNA vs. *NLRC4*-siRNA_ < 0.001; 72 h: *p*_CLP vs. *NLRC4*-siRNA_ < 0.001; *p*_NC-siRNA vs. *NLRC4*-siRNA_ = 0.002). These findings suggested that *NLRC4* gene silencing inhibited DC proliferation. Immunofluorescence (Figure 5E) was adopted to label the DC surface marker, CD86, on DCs and the results verified that the isolated cells were DCs. Moreover, the expression of the NLRC4 was effectively knocked down in the *NLRC4*-siRNA group compared with NC-siRNA group.

### Repression of NLRC4 prevents cell cycle entry and increases the DC apoptosis rate in mice with septic shock

PI single staining and AnnexinV-FITC/PI double staining methods were finally adopted to observe changes in the DC cell cycle distribution post-transfection with different vectors. The results of PI single staining revealed that when compared with the sham group, the percentage of cells arrested at the G1 phase was notably reduced (shortened G1 phase), whereas the percentages of cells arrested at the G2 and S phase were significantly elevated (prolonged G2 and S phases) in the CLP and NC-siRNA groups (all *p*_sham vs. CLP_ < 0.001; *p*_sham vs. NC-siRNA_ < 0.001). No significant differences were detected in regards to cell cycle distribution in G1 (*p*_sham vs. *NLRC4*-siRNA_ = 0.663), G2 (*p*_sham vs. *NLRC4*-siRNA_ = 0.640) or S phase (*p*_sham vs. *NLRC4*-siRNA_ = 0.999) in the *NLRC4*-siRNA group. In comparison with the CLP and NC-siRNA groups, whereas the percentage of cells arrested at the G1 phase was elevated (prolonged G1 phase) and the percentages of cells arrested at the G2 and S phases were reduced (shortened G2 and S phases) in the *NLRC4*-siRNA group (all *p*_CLP vs. *NLRC4*-siRNA_ < 0.001; *p*_NC-siRNA vs. *NLRC4*-siRNA_ < 0.001).

**Figure 6:**
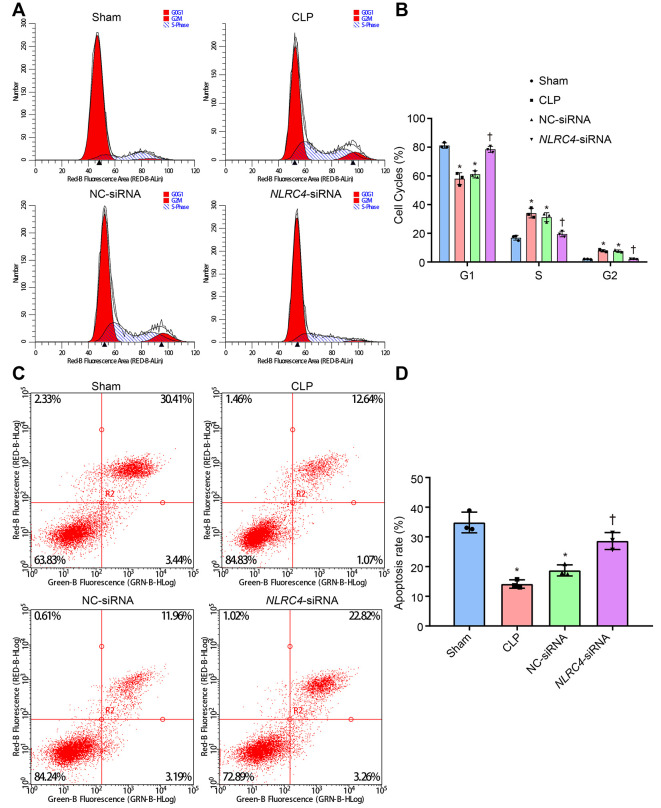
**siRNA-mediated silencing of *NLRC4* elevates DC cell apoptosis rate in mice with septic shock as evidenced by PI single staining and AnnexinV-FITC/PI double staining.** (**A**, **B**), DC cycle distribution in the sham, CLP, NC-siRNA, and *NLRC4*-siRNA groups detected by PI single staining. (**C**, **D**) DC apoptosis in the sham, CLP, NC-siRNA, *NLRC4*-siRNA groups revealed by AnnexinV-FITC/PI double staining. The bone marrow-derived DCs from the same group of mice were adopted in the *in vivo* experiments. * *p* < 0.05 vs. the sham group. † *p* < 0.05 vs. the CLP and NC-siRNA groups. Data comparison among multiple groups was analyzed by one-way ANOVA, followed by Tukey’s post hoc test. The experiment was repeated 3 times independently.

In addition, AnnexinV-FITC/PI double staining results indicated that the DC apoptotic rate was lower in the CLP (14.14 ± 1.41%) and NC-siRNA (18.74 ± 1.87%) groups when compared with the sham group (34.87 ± 3.48) (all *p*_sham vs. CLP_ < 0.001; *p*_sham vs. NC-siRNA_ < 0.001), with no significant differences observed in the *NLRC4*- siRNA group (28.63 ± 2.86%) (*p*_sham vs. *NLRC4*-siRNA_ = 0.066). DC apoptotic rate was elevated in the *NLRC4*-siRNA group (*p*_CLP vs. *NLRC4*-siRNA_ < 0.001; *p*_NC-siRNA vs. *NLRC4*-siRNA_ = 0.006) when compared with the CLP and NC-siRNA groups. These results suggested that *NLRC4* gene silencing increased the DC apoptotic rate among mice with septic shock (Figure 6).

## DISCUSSION

Septic shock is a common complication among patients in the ICU, which can lead to various organ failures and deaths [16]. A large array of cytokines are released as a result of systemic sepsis, with TNF-α being a crucial cytokine implicated in the induction of septic shock [17]. Studies have identified that *NLRP3*, a member of the NLR family is induced by TNF-α based on its own induction of Caspase-1 activation [18]. With the aim of exploring the relationship between the NLR family and septic shock, we investigated the effects of the *NLRC4* gene in mice models of septic shock. Our findings suggested that the silencing of *NLRC4* could alleviate septic shock-induced inflammatory reactions and lung tissue injury, as well as DC-mediated immune response by negatively mediating the NLR pathway.

Our results initially demonstrated that *NLRC4* silencing significantly reduces the degree of lung tissue injury induced by septic shock in mice models. The elevated expression of NLRC4 was observed in septic patients compared to that of healthy individuals [19]. Consistently, CA has been previously reported to improve the survival rate of septic shock mouse models, accompanied by inhibited inflammasome activation including NLRC4 [20]. NLRs are intracellular immunosensors associated with pathogen and damage associated molecular patterns that have unique roles in lung antibacterial immune responses during bacterial infection [21, 22]. In addition, *NLRC4* has been implicated in the process of injury and inflammation during brain ischemia, thus serving as a potential treatment target for various pathological conditions [23]. The available evidence and our results suggest that *NLRC4* silencing could potentially alleviate lung tissue injury induced by septic shock.

Our findings also highlighted that *NLRC4* silencing could negatively regulate the NLR pathway. *NLRC4* is a member of the NLR family, which are cytosolic receptors that target bacterial molecules [24]. These NLRs participate in a variety of innate immune pathways, including the adjustment of the NF-κB pathway for *NOD1* and *NOD2*, and the assembling of complexes for NLR proteins NLRP1, NLRC4, and NLRP3 [25]. Substantial evidence has been indicated that *NLRP3* and *NLRC4* inflammasomes possess well-characterized protective functions in alcoholic-induced liver injury [25]. *NOD1* and *NOD2* are recognition receptors related to cytosolic patterns, which are important for inherent immune signaling [26]. Moreover, *NOD1* and *NOD2* were discovered to share a relation with *RIP2*, which is a downstream signaling molecule receptor [27]. The activation of NOD1 results in septic shock and various organ injury/dysfunction in animal models [28]. Our findings illustrated that silencing *NLRC4* resulted in significant reductions in the expressions of the NLR pathway key players including *NLRC4*, *NOD1*, *NOD2*, and *RIP2*. This suggests *NLRC4* silencing was able to negatively regulate the NLR pathway.

Furthermore, we identified that *NLRC4* silencing could attenuate inflammatory reactions induced by septic shock. TNF-α, IL-1β, and IL-6 are all well-known pro-inflammatory cytokines as previously reported [29]. Studies have also demonstrated that the *NLRC4* inflammasome functions in the regulation and release of pro-inflammatory cytokines in response to an array of microbial stimuli [8]. The macrophage *NLRC4* inflammasome initiates potent innate immune responses against Salmonella by eliciting caspase-1-dependent pro-inflammatory cytokine production [30]. A previous study elucidated that *NLRP3* and *NLRP4* work to regulate production and pyroptosis of IL-18 and IL-1β, thus testifying that *NLCR4* silencing causes the inhibition of IL-1β, which is in accordance with our findings [31]. A prior study also noted that gentiopicroside conferred protection against shock and decreased inflammatory cytokine production of IL-1β, IL-6 and TNF-α in lung tissues [32]. Given the above key findings, it is reasonable to regard silenced *NLRC4* as an important gene when inhibiting inflammatory reactions.

Lastly, *NLRC4* gene silencing was observed to suppress the maturation and proliferation of DCs, while promoting DCs apoptosis. DCs are widely recognized as specialized antigen-presenting cells which begin maturing upon coming in contact with antigens via recognized receptors [33]. Elevated DCs have been identified in the colonic mucosa of patients suffering from Crohn’s disease, another chronic inflammatory bowel disease [34]. Previous studies have further confirmed that *NLRC4* promotes the cleavage and maturation of pro-inflammatory cytokines [24]. In a previous study, the activation of *NLRC4* inflammasome has been documented in splenic DCs [35]. We speculated that *NLRC4* silencing could inhibit maturation of DCs as well as inhibit their population. *NLRC3* has been proposed to inhibit the processes related to colorectal cancer through the regulation of cellular proliferation and apoptosis levels [21]. A functional study demonstrated that *NLRC5* gene silencing inhibits TGF-β1-induced proliferation, while promoting the apoptosis of LX-2 cells [36]. We acquired the basic understanding of the relationship between *NLRC4* gene silencing and maturation, proliferation, and apoptosis of DCs, in accordance with the investigated literature as well as our obtained results.

**Figure 7:**
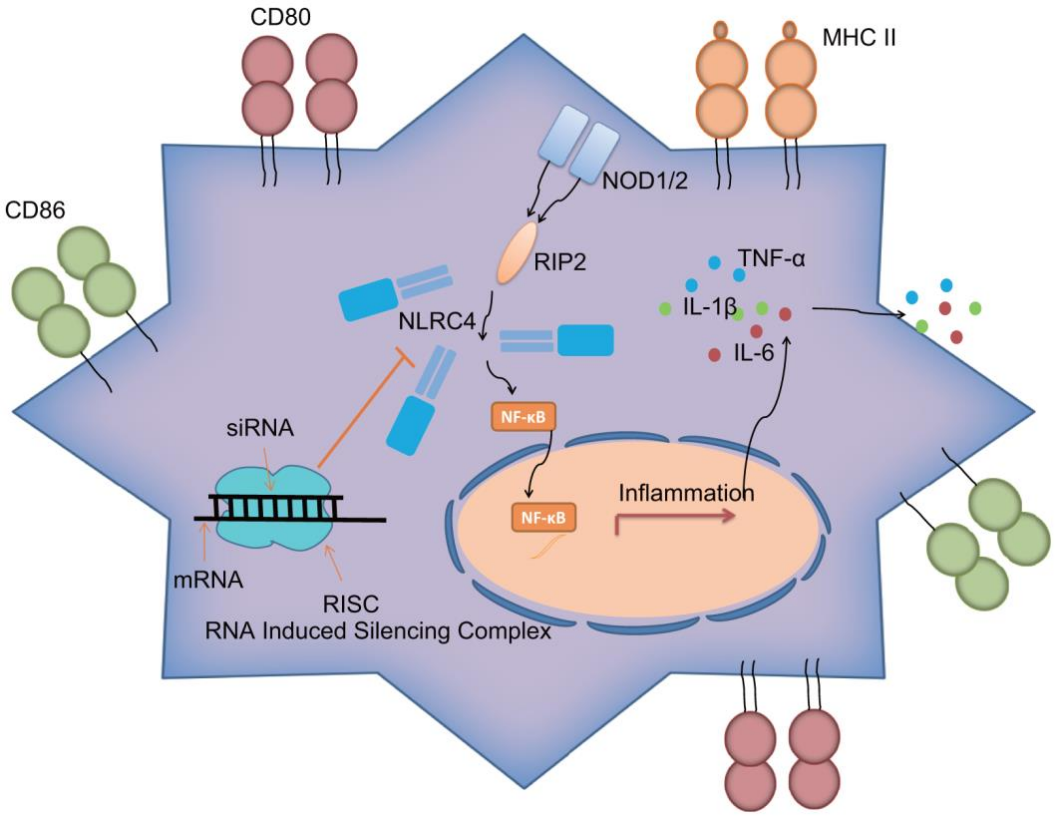
***NLRC4* gene regulates inflammatory reaction and immune response of DCs in septic shock by mediating the NLR pathway.** *NLRC4* is the receptor of the NLR pathway. Activated NF-κB pathway induces inflammatory reaction with secretion of inflammatory factors: IL-1β, TNF-α and IL-6 outside cells, as well as increases in CD80, CD86 and MHC II on the cytomembrane, thus activating DC immune response. Importantly, siRNA-mediated silencing of *NLRC4* blocks the NLR signaling pathway to inhibit DC maturation and immune response, which alleviates the lung tissue injury of mice induced by septic shock.

Overall, the current study demonstrated that siRNA-mediated silencing of *NLRC4* blocks the NLR signaling pathway to inhibit DC maturation and immune response, which consequently alleviates lung tissue injury induced by septic shock (Figure 7). However, due to limited time and funding, we solely relied on a single technique (siRNA) to inhibit *NLRC4* in our study, highlighting the need for alternative methods such as the use of *NLRC4* knockout mice and/or pharmacological inhibitors or transient introduction of cas9 to knock down NLRC4 *in vitro* to validate the current findings, which will be carried out in our future ventures. Moreover, further studies of larger sample sizes would be conducted.

## MATERIALS AND METHODS

### Ethics statement

All animal experiment protocols were approved by the Ethics Committee of the Second Affiliated Hospital of Nanchang University and conducted in strict accordance with the recommendations in the Guide for the Care and Use of local Laboratory Animals. All efforts were made to minimize the number and suffering of the included mice in the study.

### Experimental animals

Forty healthy C57BL/6 male mice (age: 6 - 8 weeks; weight: 18 - 22 g) were purchased from the Laboratory Animal Center of Jiangxi University of Traditional Chinese Medicine (Nanchang, Jiangxi, China).

All procured mice were raised under specific pathogen-free conditions at a room temperature of 23° C with a relative humidity of 65%, following 12-h light-dark cycles. The mice were not fed but were allowed free access to water for a period over 12 h. Septic models were established in 30 mice by CLP as previously described [37]. Meanwhile, 10 mice were sham-operated with cecum without ligation and puncture. The 30 CLP mice were then assigned into three subgroups: CLP (mice with CLP-induced septic shock), negative control (NC)-siRNA (CLP mice injected with NC-siRNA plasmid), and *NLRC4*-siRNA (CLP mice injected with *NLRC4*-siRNA plasmid) groups (n = 10/group). Based on the known sequence of *NLRC4* in the National Center for Biotechnology Information, the NC-siRNA plasmid and *NLRC4*-siRNA plasmid were constructed by Sangon Biotechnology Co., Ltd. (Shanghai, China) using the pCDNA3.1 vector (Shanghai GenePharma, Co., Ltd., Shanghai, China). The sequence of the NC-siRNA was 5’-TACGTCCAAGGTCGGGCAGGAAGA-3’, while the sequence of *NLRC4*-siRNA was 5’-TACGTCCAAGGTCGGGCAGGAAGA-3’. Then, 0.1 mL recombinant retroviral vector (1.95 × 10^8^ PFU) expressing NC-siRNA (scrambled siRNA) or *NLRC4*-siRNA was injected into the successfully modeled mice via the tail vein 2 h after CLP surgery [38, 39]. The mice were then euthanatized 24 h after CLP surgery with their lungs promptly collected under sterile conditions. Parts of the lung tissues were fixed in 4% formalin for HE staining, while the remaining were stored in liquid nitrogen for further use in the following procedures: RT-qPCR, Western blot analysis and ELISA. The femur medullary cavity of all mice was collected following the aforementioned aseptic procedures for collection of bone marrow-derived DCs for further use.

### HE staining

The formalin-fixed lung tissues were rinsed with tap water 72 h later, routinely dehydrated for 1 min/time, permeabilized with xylene twice (5 min/time), soaked in wax, placed in a paraffin mold, and cooled in a paraffin embedding machine. The embedded tissue pieces were sectioned (5μm) on a microtome before the sections were dried at 70° C for 1 h, and heated at 60° C for 5 h. The paraffin sections were dewaxed to liquid form and subsequently stained with hematoxylin at room temperature for 10 min. The sections were rinsed by running water for 30-60 s, differentiated using 1% hydrochloric acid alcohol for 30 s, and then rinsed under running water for 5 min. The sections were stained with eosin (0001-H, Beijing Xinhualvyuan Science and Technology Ltd., Beijing, China) at room temperature for 1 min [40], dehydrated using varied concentrations of alcohol solutions (concentrations of 70%, 80%, 90%, 95%, and 100%, respectively; 1 min/time), and treated with xylene phthalate before the sections were permeabilized with xylene I and II (GD-RY1215-12, Shanghai, China; 1 min/time) twice and finally mounted using neutral gum. Subsequently, the lung tissues were observed and photographed with a Cai Si fluorescence microscope (PrimoStar iLED; Bioresearch Technology Co., Ltd., Beijing, China). The lung tissue injury scores were evaluated based on the Mikawar method by assessing alveolar congestion, hemorrhage, infiltration or aggregation of neutrophils in airspace or vessel wall, and the thickness of alveolar wall hyaline membrane formation. These items were scored according to the following: 0 minimal damage, 1 mild damage, 2 moderate damage, 3 severe damage, and 4 maximal damage [41].

### RT-qPCR

A total of 100 μL prepared frozen lung tissue homogenate was collected and added with l mL TRIzol reagents (15596-018, Beijing Solarbio Science and Technology Co., Ltd., Beijing, China) in order to extract the total RNA content from the frozen tissues. Next, the concentration and quality of the extracted total RNA were determined using a NanoDrop 2000 spectrophotometer (Thermo Fisher Scientific, Rockford, IL, USA). When the ratio of 260/280 was about 2.0 and concentration was 1 μg - 5 μg, the RNA was used for the follow-up experiments. The RNA (2 μg) was then reverse transcribed into cDNA using TaqMan Reverse Transcription Reagents (Roche Ltd., Basel, Switzerland) at 42° C for 50 min. Gene fragments of *NOD1*, *NOD2*, *NLRC4*, receptor-interacting protein 2 (*RIP2*) and Nuclear factor-kappaB (*NF-κB*) were amplified using PCR. The primer sequences for RT-qPCR were synthesized by Sigma-Aldrich Chemical Company (St Louis, MO, USA) (Table 2). PCR amplification was conducted in real-time quantitative PCR instrument (CFX96, Bio-Rad Laboratories, Hercules, CA, USA). With GAPDH as the internal reference, the relative transcript level of target gene mRNA was calculated using the 2^-ΔΔCT^ method [42].

**Table 2:** Primer sequences for RT-qPCR.

**Gene**	**Primer sequence (5' - 3')**
*NOD1*	F: 5'- ACTCAGCG-TCAACCAGATCAC-3'
	R: 5'-ACGATGGAGGTGCTGTTCTTC-3'
*NOD2*	F: 5'-CTCAGTCTCGCTTCCTCAGTAC-3'
	R: 5'-TGCAGA-AGAGTGCTCTTGCC-3'
*NLRC4*	F: 5'-ATCGTCATCACCGTGTGGAG-3'
	R: 5'-GCCAGACTCGCCTTCAATCA-3'
*RIP2*	F: 5'-TCCAGAGTAAGAGGGAAGCC-3'
	R: 5'-TTGGATGTCAGACGTATCTAGC-3'
*NF-κB*	F: 5'-CCT CTGGCGAATGGCTTTAC-3'
	R: 5'-GCTATGGAT ACTGCGGTCTGG-3'
*GAPDH*	F: 5'-TTCACCACCATGGAGAAGGC-3'
	R: 5'-GGCATGGACTGTGGTCATGA-3'

Note: RT-qPCR, reverse transcription quantitative polymerase chain reaction; *NOD1*, nucleotide binding and oligomerization domain-1; *NOD2*, nucleotide binding and oligomerization domain-2; *NLRC4*, NLR family CARD domain containing 4; *RIP2*, receptor-interacting protein 2; *NF-κB*, Nuclear factor-kappaB; *GAPDH*, glyceraldehyde-3-phosphate dehydrogenase; F, forward; R, reverse.

### Western blot analysis

First, 100 μL of the prepared frozen lung tissue homogenate was extracted and placed into reaction tubes, which were then added with 1 mL of cell lysis solution and left to react 30 min at 4° C, with a shaking every 10 min. Next, the sample was centrifuged at 12000 r/min and 4° C for 20 min and the lipid layer was discarded. The supernatant was collected as the protein extraction solution. A bicinchoninic acid kit (20201ES76, YEASEN Biotechnology Co., Ltd., Shanghai, China) was used to determine the concentration of the extracted protein. The total protein was subsequently treated with sodium dodecyl sulfate polyacrylamide gel electrophoresis and transferred onto a nitrocellulose membrane. The membrane was then blocked with 5% skimmed milk powder at room temperature for 1 h and subsequently probed with the following primary antibodies (Abcam Inc., Cambridge, MA, USA): rabbit anti-mouse NLRC4 (ab201792, 1 : 1000), NOD1 (ab22143, 1 : 1000), NOD2 (ab124348, 2 μg/mL, 1 : 1000), anti-RIP2 (ab8428, 1 : 1000), anti-NF-κB (ab28856, 1 : 1000) and glyceraldehyde-3-phosphate dehydrogenase (GAPDH) (ab181602, 1 : 10000) at 4° C overnight. The membrane was later rinsed with Tris-Buffered Saline Tween-20 (TBST) and re-probed with the diluted horseradish peroxidase-labeled goat anti-rabbit immunoglobulin G (IgG) and left at room temperature for 1 h. After developing, the gray values of each protein band in the membrane were analyzed using the Quantity One v4.6.6 software, followed by the quantitative analysis of proteins (GAPDH as the internal reference) [43].

### ELISA

A total of 100 μL of frozen lung tissue homogenate was extracted and transferred to a reaction tube. The expressions of IL-1β, TNF-α and IL-6 in lung tissues of mice were detected in strict accordance with the manual of mouse IL-1β, TNF-α, and IL-6 ELISA kits (Wuhan Zhongzhi Biotechnologies Ltd., Wuhan, Hubei, China), respectively [44].

### Isolation and culture of DCs

The separated femur was repeatedly washed and cleaned until the white part of the bone could be seen, followed by the collection of the bone marrow. The bone marrow was centrifuged at 256 × g for 5 min. After the removal of the supernatant, the bone marrow cells were obtained from the precipitate at the bottom of the tube, added with erythrocyte lysate (1 : 10, preheated at 37° C), and mixed thoroughly. The mixture was then left to sit at room temperature for 2 - 4 min, followed by the removal of excess red blood cells. The bone marrow cells were subsequently suspended in Roswell Park Memorial Institute (RPMI) 1640 medium (Wuhan Alpha Biotechnologies Co., Ltd., Wuhan, Hubei, China) containing 10% fetal calf serum (FCS) (Hangzhou Sijiqing Company, Zhejiang, China) and adjusted to a suspension of 2 × 10^6^ cells/mL. The suspension was then placed into a glass cell culture flake and incubated at room temperature for 2 - 3 h. After removing detached cells, the concentration of the bone marrow cell suspension was readjusted to 1 × 10^8^ cells/L using RPMI 1640 complete medium (sodium pyruvate, Glutamine, Hepes, and NaHCO_3_ at a ratio of 1 : 2 : 10 : 8.9), penicillin, streptomycin (200,000 μ/L each), GM-CSF, IL-4 (50 ng/L each, Yarewell Technology Inc., Shenzhen, China), mercaptoethanol (50 μmol/L) and FCS (20%). Afterwards, the cell suspension (1.5 - 2 mL per well) was added to a 6-well plate for incubation with 5% CO_2_ at 37° C. Half of the culture medium was renewed every other day, and the non-adherent cells were removed at the 3^rd^ h and on the 3^rd^ d following incubation. The cell incubation condition was observed on the 5^th^ - 7^th^ days, while the morphology and quantity of DCs were observed on the 3^rd^ h, 3^rd^ d, 5^th^ d, and 7^th^ d under an inverted microscope. After 7 d of incubation, DCs were collected for further experimentation [45].

### Immunofluorescence

Cells (2 × 10^4^ cells/well) were cultured overnight. When the cells reached 50-70% confluence, they were washed twice with PBS, fixed in a 4% paraformaldehyde and permeabilized in a 0.03% Triton X-100 (Sigma) PBS solution for 20 min. Cells were then washed 3 times with PBS for 5 min each and blocked with 5% bovine serum albumin (BSA) in PBS for 1 h at room temperature. Cells were incubated with their corresponding primary antibodies CD86 (1: 100; Abcam) and NLRC4 (1: 100; Abcam) in a humidified cabinet overnight at 4° C. The cells were later washed 3 times in PBS for 5 min each and incubated with a CY3-conjugated secondary antibody (1: 50; CWBIO, Beijing, China) at room temperature for 1 h in the dark. Finally, the cells were washed three times in PBS and incubated with 1 μg/ml of 4,6-diamidino-2-phenylindole (DAPI, Roche, Basel, Switzerland) for 5 min at room temperature in the dark. The slides were thoroughly washed with PBS, and observed with an inverted fluorescence microscope (400 ×; Nikon, Tokyo, Japan) [46].

### Immunohistochemistry

Immunohistochemical staining of F4/80, CD45 were performed adhering to instructions provided by UltraSensitive™ SP Kit (KIT-9710, Maxim Bio, Fuzhou, China). The primary antibodies used were as follows: anti-CD45 (1: 8000, Abcam), and anti-F4/80 (GB11027, 1: 1000, Servicebio, Wuhan, China). Sections were incubated with non-immune serum instead of the primary antibody that was designated as NCs. Images were analyzed with Image-Pro Plus 5.1. software (Media Cybernetics, Inc., Rockville, MD, USA). Immunoactivity of F4/80 and CD45 were quantified with the percent of positive cells [46].

### Flow cytometry

After culture for 7 d, DCs were obtained from the mice in each group and detached with 0.25% trypsin to harvest the adherent cells. DCs were then dispersed into a single-cell suspension, re-suspended with 100 μL PBS and incubated with the following Fluorescein isothiocyanate (FITC)-labeled monoclonal antibodies: FITC-labeled anti-mouse CD80 (0.5 μL), and CD86 (0.5 μL) (BioLegend Inc., San Diego, CA, USA) and FITC-labeled anti-mouse I-Aα (0.5 μL) (BD Pharmingen Inc., San Diego, CA, USA) at 4° C for 30 min without any exposure to light. The supernatant was removed after centrifugation and the cells were rinsed with 1 mL PBS and re-suspended with 0.5 mL PBS at 4° C without any exposure to light. Finally, the cells were detected using a flow cytometer (BD Pharmingen Inc., San Diego, CA, USA) and analyzed with the CellQuest 5.1 software [47].

### MTT assay

DCs were seeded in 96-well plates (3 × 10^3^ - 6 × 10^3^ cells/well) at a cell volume of 0.2 mL in each well, with 6 duplicate wells set up for each group. At the 24^th^ h, 48^th^ h, and 72^th^ h time periods of incubation, the culture plates were further cultured for another 4 h with 5 g/L 10% MTT solution (GD-Y1317, Shanghai Guduo Bio-technology Co., Ltd., Shanghai, China). Dimethyl sulfoxide (D5879-100ml, 100 μL, Sigma-Aldrich Chemical Company, St Louis, MO, USA) was added to each well and gently shaken for 10 min to dissolve the formazan crystals. The OD value of each well at an excitation wavelength of 570 nm was measured using a microplate reader (BS-1101, Detie Lab, Nanjing, Jiangsu, China). Subsequently, a cell viability curve was plotted with time as the abscissa and OD value as the ordinate [48].

### AnnexinV-FITC/PI staining

The cell cycle distribution of DCs was analyzed using PI single staining. After 7-d incubation, the DCs were collected, washed 3 times with cold PBS and centrifuged. After the removal of the supernatant, the DCs were re-suspended with PBS with the cell concentration adjusted to 1 × 10^5^ cells/mL, fixed in pre-cooled 70% ethanol solution and incubated at 4° C overnight. With the supernatant removed through centrifugation at 800 × g at 4° C, the cells were rinsed twice with PBS containing 1% fetal bovine serum, re-suspended in 400 μL binding buffer and incubated with 50 μL RNase A (R4875, Wegene Bio-Technology Co., Ltd., Shanghai, China) at 37° C for 30 min. A total of 50 μL PI (50 mg/L) (GK3601-50T, Beijing Dingguo Changsheng Biotechnology Co., Ltd., Beijing, China) was added for 30-min incubation devoid of light. A flow cytometry was later performed to detect the cell cycle distribution.

AnnexinV-FITC/PI double staining methods were applied to assess DC cell apoptosis. After 7 d of incubation, DCs were obtained, detached with ethylenediaminetetraacetate-free 0.25% trypsin (YB15050057, Yubo Biological Technology Co., Ltd., Shanghai, China), collected into a flow tube, and centrifuged, followed by removal of the supernatant. The cells were then rinsed 3 times with cold PBS, and centrifuged with the supernatant discarded. Annexin-V-FITC/PI dye liquor [Annexin-V-FITC : PI : N-(2-hydroxyethyl) piperazine-N'-2-ethanesulfonic acid (HEPES) = 1 : 2 : 50] was prepared in accordance to the instructions of the Annexin V-FITC apoptosis kit (K201-100, BioVision, CA, USA). Every 100 μL dye liquor was used to resuspend 1 × 10^6^ cells, incubated at room temperature for 15 min and uniformly mixed with 1 mL HEPES buffer solution (PB180325, Procell, Wuhan, China). DC cell apoptosis was then analyzed at wavelengths of 488 nm as well as 525 and 620 nm bandpass filter to detect FITC and PI fluorescence [49].

### Statistical analysis

Data analyses were performed using the SPSS 19.0 software (IBM Corp., Armonk, NY, USA). Measurement data were expressed as mean ± standard deviation of at least three independent experiments. Comparisons among multiple groups were analyzed by one-way analysis of variance (ANOVA) with Tukey's tests. The repeated measures ANOVA with Bonferroni corrections was applied for the comparison of data at different time points. A *p* value of < 0.05 was indicative of statistical significance.

## Supplementary Material

Supplementary Figure 1
