# Supplementary material for: NLRC4 gene silencing-dependent blockade of NOD-like receptor pathway inhibits inflammation, reduces proliferation and increases apoptosis of dendritic cells in mice with septic shock
Source: Aging (Albany NY). 2021 Jan 6;13(1):1440–57. doi: 10.18632/aging.202379 (PMC7835030; doi:10.18632/aging.202379)
Supplement: Supplementary Figure 1 [file aging-13-202379-s001.pdf]

SUPPLEMENTARY FIGURE

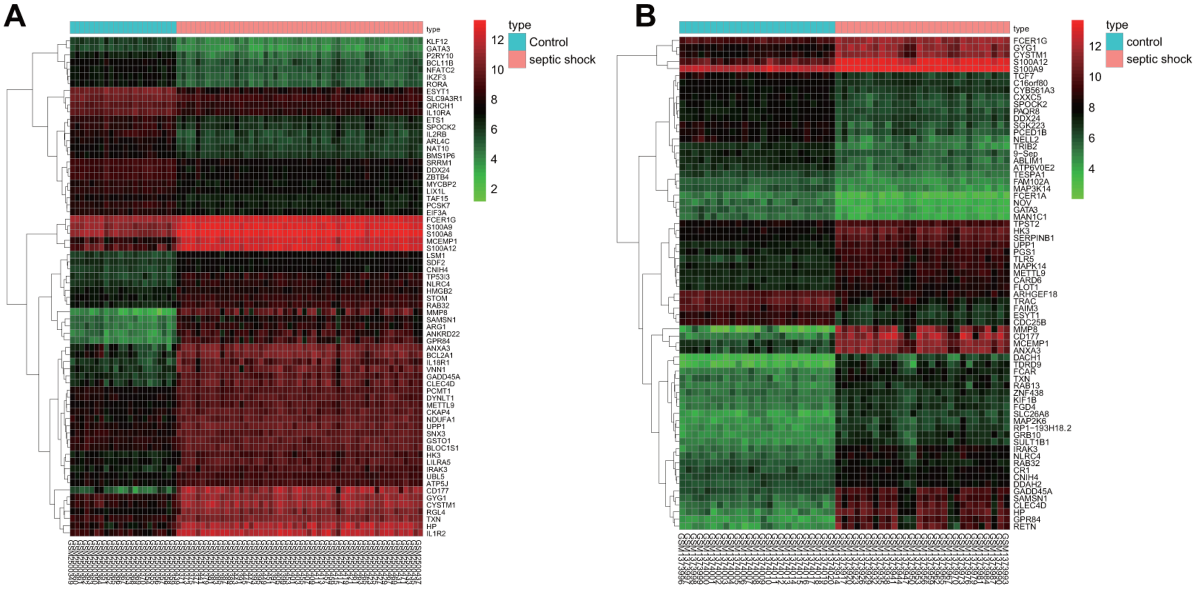

Supplementary Figure 1. Heatmaps illustrating the DEGs in microarray data GSE95233 and GSE57065 related to septic shock. (**A**, **B**) the former 70 DEGs in the microarray expression profiles GSE95233 and GSE57065 (the abscissa stands for the sample number; the ordinate refers to the DEGs; the histogram in upper right is the color gradation; each rectangle in the figure refers to a sample expression value).
